# Editorial: Non-chemical strategies for managing plant diseases and abiotic stresses

**DOI:** 10.3389/fpls.2026.1920616

**Published:** 2026-07-23

**Authors:** Charith Raj Adkar-Purushothama, Ramu S. Vemanna, Kirankumar S. Mysore

**Affiliations:** 1 RNA Group/Groupe ARN, Département de Biochimie et de génomique fonctionnelle, Faculté de Médecine et des Sciences de la Santé, Pavillon de Recherche Appliquée au Cancer, Université de Sherbrooke, Sherbrooke, QC, Canada; 2 Regional Center for Biotechnology, Faridabad, Haryana, India; 3 Department of Biochemistry and Molecular Biology, Oklahoma State University, Stillwater, OK, United States

**Keywords:** biological control, biopesticides, host resistance and tolerance, integrated pest management (IPM), RNAi - RNA interference

The increasing frequency of plant disease outbreaks and abiotic stresses poses a significant threat to global agricultural productivity and food security. Climate change, intensive agricultural practices, and the emergence of new pathogen populations have intensified the need for sustainable crop protection strategies. While chemical pesticides remain important tools for disease management, concerns about environmental contamination, non-target effects, and resistance development have spurred growing interest in non-chemical approaches. These strategies include biological control, host resistance, microbiome-based interventions, ecological management, and innovative breeding approaches that enhance plant resilience while reducing dependence on synthetic inputs.

This Research Topic, *Non-chemical Strategies for Managing Plant Diseases and Abiotic Stresses*, was established to highlight recent advances in sustainable approaches for managing plant diseases and improving crop resilience. The collection brings together original research and review articles addressing multiple dimensions of non-chemical disease management, ranging from biological control and host resistance to integrated breeding strategies and One Health perspectives for crop plants.

A recurring theme across the contributions is the importance of harnessing biological processes and ecological interactions to suppress plant diseases. Du et al. demonstrated how multi-omics approaches can be used to unravel the mechanisms underlying the biological suppression of tomato bacterial wilt. Their work revealed complex interactions among microbial communities, host responses, and pathogen suppression processes that contribute to disease control. These findings advance our understanding of the mechanistic basis of biological control and highlight the importance of systems-level investigations for developing reliable and effective microbe-based disease management strategies.

The role of biological control is further explored by Mourou et al. in their review about current strategies for managing *Xylella fastidiosa*. As one of the most destructive bacterial pathogens affecting perennial crops worldwide, *X. fastidiosa* presents substantial challenges for sustainable disease management. The current knowledge on microbial antagonists, endophytes, and other biological interventions is discussed in the review. In addition, they also discuss practical limitations and opportunities for implementation. Their review emphasizes the need to integrate biological control within broader disease management frameworks to achieve durable pathogen suppression.

Host resistance remains one of the most effective and environmentally sustainable approaches for disease management. Konigopal et al. investigated the intrinsic host range of *Meloidogyne enterolobii* and virulent populations of *M. incognita*. Their findings provide valuable insights into host susceptibility and resistance, supporting crop rotation planning, resistance deployment, and nematode management programs. As virulent root-knot nematode populations continue to expand globally, such knowledge will be important for designing durable non-chemical control strategies.

Advances in crop improvement reported by Iqbal et al., proposed the concept of Breeding for Integrated Pest Management (B-IPM). This framework advocates the development of crop varieties that not only possess resistance traits but also enhance the effectiveness of biological control agents and other integrated pest management components. By linking plant breeding with ecological pest management, the authors present a holistic strategy for improving crop protection while reducing reliance on chemical inputs. The B-IPM concept illustrates how future breeding programs may contribute to sustainable agricultural intensification.

The importance of accessible and locally adapted disease management approaches is highlighted by Verma et al., who evaluated organic vinegar treatments and black gram genotypes for managing pod rot disease caused by *Fusarium humuli*. Their study demonstrates the potential of combining host resistance with low-input strategies that can be readily adopted across diverse production systems. Such approaches are particularly valuable in resource-limited agricultural settings, where affordable and environmentally compatible alternatives are urgently needed.

Pame et al. developed soil-free bioassays for evaluating novel control agents against *Phytophthora cinnamomi* root rot. Their study highlights the potential of RNA-based biopesticides and innovative assay systems for advancing environmentally sustainable disease management, further reinforcing the importance of integrating cutting-edge non-chemical strategies into crop management systems and enhancing plant resilience under controlled and field conditions.

The broader implications of sustainable disease management are addressed by Adkar-Purushothama et al., who reviewed non-chemical strategies for controlling fungal pathogens from a One Health perspective. Their work highlights the interconnectedness of plant, environmental, and human health and synthesizes advances in biological control, host resistance, induced resistance, and ecological management. Importantly, the authors argue that disease management strategies should be evaluated not only for their efficacy but also for their contribution to environmental sustainability and ecosystem health.

Collectively, the articles in this Research Topic demonstrate the diversity and growing sophistication of non-chemical approaches to manage plant diseases. Despite differences in pathosystems, methodologies, and production contexts, several common themes emerge. First, effective disease management increasingly depends on understanding complex interactions among plants, pathogens, beneficial microorganisms, and the environment. Second, durable solutions are likely to require the integration of multiple complementary approaches rather than reliance on a single intervention. Third, advances in molecular biology, microbiome research, and breeding technologies are creating new opportunities for developing sustainable disease management strategies adaptable across agricultural systems.

The contributions assembled here also highlight important future research directions. Continued efforts are needed to improve the consistency and scalability of biological control agents under field conditions, deepen our understanding of microbiome-mediated disease suppression, and accelerate the deployment of resistance traits through modern breeding approaches. Furthermore, interdisciplinary collaborations integrating plant pathology, microbial ecology, genomics, breeding, and agronomy will be essential for translating scientific discoveries into practical solutions.

As modern agriculture seeks to balance productivity with environmental stewardship, non-chemical strategies will play an increasingly important role in crop protection. The studies presented in this Research Topic provide valuable insights into the mechanisms, opportunities, and challenges of sustainable disease management, contributing to the growing body of knowledge that supports resilient agricultural systems. Together, these contributions reinforce the view that non-chemical approaches are becoming central components of future crop protection systems and sustainable agriculture.

